# Functional Not Medical Frailty Is Associated With Long-Term Disability After Surgery for Colorectal Cancer

**DOI:** 10.7759/cureus.23216

**Published:** 2022-03-16

**Authors:** Meridith Ginesi, Katherine Bingmer, Johnathan T Bliggenstorfer, Asya Ofshteyn, Emily Steinhagen, Sharon L Stein

**Affiliations:** 1 Department of Surgery, University Hospitals Research in Surgical Outcomes and Effectiveness (UH RISES), Cleveland, USA; 2 Department of Colorectal Surgery, University Hospitals Cleveland Medical Center, University Hospitals Research in Surgical Outcomes and Effectiveness (UH RISES), Cleveland, USA

**Keywords:** colorectal cancer, medical frailty, functional frailty, disability, colorectal surgery

## Abstract

Background: Frailty has been associated with increased morbidity after surgery. However, few studies investigate long-term functional outcomes.

Methods: Patients ≥ 65 years old who underwent surgery for colorectal cancer were surveyed regarding their ability to perform activities of daily living, measured by Barthel Index, before and after surgery. Patients also reported time to return to their functional baseline.

Results: Pre-operative moderate dependency was associated with declining function at six months (OR: 8.8; CI: 1.8-42.6) and one year post-operatively (OR: 17.5; CI: 2.8-109.8). Pre-operative functional frailty was associated with subjective failure to return to baseline (OR: 4.8 and 4.2) for slightly and moderately dependent patients and a longer time to return to baseline. Medical frailty, based on the modified Frailty Index, was not significantly associated with failure to return to baseline.

Conclusions: Measures of functional frailty are better predictors of failure to return to baseline, than measures of medical frailty.

## Introduction

Colorectal cancer is frequently a disease of the elderly, with 60-70% of all new diagnoses occurring in patients over 65 years of age [1]. As the number of geriatric patients in the United States grows, clinicians will be more frequently called upon to care for these patients and their comorbidities. Medical frailty, a condition of physiological decline, is prevalent among this patient population and has been demonstrated to significantly increase the risk for mortality, and other 30-day post-operative complications [1]. As a result, frailty and its effects on patient outcomes have become an area of interest for surgical research. While most of the literature focuses on short-term outcomes, restitution of baseline function over the long-term has been shown to be a high priority for older patients and is poorly studied [2,3].

Prior literature on frailty and surgical outcomes is limited in two major ways: a focus that is limited to short-term outcomes, and studies that limit the assessment of frailty to medical comorbidities. There is a significant body of literature that demonstrates frailty’s association with negative post-operative outcomes, but often limits evaluations to medical frailty and short-term outcomes including length of stay, post-operative serious and minor complications, discharge to rehab or skilled nursing facility, hospital readmission, and mortality [4-9]. Studies that evaluate functional independence do show an association between both age and pre-operative function and post-operative loss of independence but are generally limited to the 30-day post-operative period [10,11]. Studies that do evaluate one-year outcomes primarily focus on mortality and neglect long-term function [12-14]. The few studies that do look at long-term functional outcomes show mixed results, with some demonstrating functional frailty as a risk factor for long-term functional decline, and others failing to show a significant association [15-20].

This study seeks to clarify the impact of pre-operative functional frailty on long-term outcomes after oncologic colon and rectal surgery. Specifically, this study evaluates whether patients returned to their pre-operative baseline or experienced declining function after surgery and whether medical or functional measures of frailty were predictive of this decline. We hypothesize that functional frailty is a better predictor of long-term outcomes than medical frailty.

## Materials and methods

A retrospective chart review was performed at a single academic institution, which identified all patients aged 65 years and older, with clinical stage I-III colorectal adenocarcinoma, who underwent an elective colon or rectal resection from 2014 to 2018. Patients with clinical stage IV cancer were excluded since the surgical management of these patients differed from stages I-III. This study was approved by the Institutional Review Board at University Hospitals Cleveland Medical Center. Patient demographics, comorbidities, oncologic, perioperative, and operative details as well as post-operative course details were collected. In 2020, all living patients were invited to participate in a telephone follow-up survey (Addendum A).

The modified Frailty Index (mFI) was used to identify patients in this group who were medically frail. The mFI is a validated frailty measure derived from the Canadian Study on Health and Aging Frailty Index that correlates to post-operative morbidity [21]. It includes 11 medical comorbidities such as diabetes, chronic obstructive pulmonary disease, and cardiac conditions. Scoring ranges from 0 (least frail) to 1 (most frail). Patients are considered medically frail based on mFI if their score is greater than or equal to 0.27. Frailty based on mFI is commonly used in research and has previously been shown to be associated with short-term post-operative outcomes.

Patients were interviewed via telephone about their ability to perform activities of daily living (ADLs) based on the Barthel Index. This measure was chosen with the help of institutional geriatricians after reviewing several measures of functional frailty due to its ease of use with patient self-report, frequent use in research, and high-quality data supporting its use [22-24]. The Barthel Index is a validated frailty measure that includes grooming, dressing, bathing, eating, using the bathroom, transfers, climbing stairs, mobility, and bladder or fecal incontinence. Patients are given points based on independent completion of these activities and points are totaled. A score of 100 indicates total independence (patient is able to do all ADLs listed above without help), 91-99 indicates slight dependency (patient needs help in one of the ADLs listed above), 61-90 indicates moderate dependency (patient needs help with a few ADLs listed above), 21-60 indicates severe dependency (patient needs help with many ADLs listed above), and a score of 0-20 indicates total dependency (patient is unable to do or needs complete help with most ADLs listed above). In patients that had a stoma following surgery, no points were deducted for bowel incontinence. Patients’ pre-operative, six-month post-operative, and one-year post-operative Barthel indices were then calculated as a measure of functional frailty. Lastly, patients were asked if they had returned to their pre-operative baseline level of functioning after surgery, and if so, how long this took.

Analysis

Respondents were compared to non-respondents to ensure that participating patients were representative of the entire institutional population. For all respondents, a pre-operative Barthel Index score was compared to scores at six and 12 months to assess whether patients experienced declining function after surgery. Patients were categorized as having declining function if their Barthel Index category decreased at all over six- and 12-months after surgery. Univariate and multivariable analyses were done for six- and 12-month time intervals to identify factors associated with declining function, with p ≤ 0.05 considered significant. Categorical variables were analyzed with the chi-square test and continuous variables with the Mann-Whitney U test.

Additionally, a univariate and multivariate analysis was done to identify factors associated with a subjective failure to return to baseline. The time to return to baseline function was compared using Kaplan-Meier curves, with log-rank evaluation for significance. Statistical analysis was done using Stata/IC 16.1 statistical software (StataCorp, College Station, TX).

## Results

Demographics

Initial chart review returned 291 patients, aged 65 years or older, who underwent elective colorectal surgery for cancer at our institution between 2014 and 2018. A total of 59 patients were confirmed deceased, and 25 declined to participate. A total of 110 patients chose to participate; the remaining 97 were unable to be reached via telephone. Of surveyed participants, 55 (50.0%) were female, and the majority identified as Caucasian (93, 84.6%). While clinical stage IV patients were excluded, three patients had metastatic disease found at the time of surgery, making them pathologic stage IV. Patients’ self-rating of pre-operative ability to perform ADLs demonstrated that the majority of participants were independent pre-operatively (61, 59.8%), 16 (15.7%) were slightly dependent, and 25 (24.5%) were moderately dependent based on Barthel Index. Cancer-related, surgery-related, and treatment information is reported in Table 1.

**Table 1:** Demographics of survey participants vs. non-participants. * Numbers are given as mean and standard deviation in parentheses.

	Surveyed group (n = 110), n (%)	Not surveyed (n = 181), n (%)	P-value
Sex			
Female	55 (50.0)	88 (48.6)	0.819
Race			
White	93 (84.6)	141 (78.3)	0.243
Black	15 (13.6)	36 (20.0)	
Other	2 (1.8)	4 (1.7)	
Age*	73.2 (5.7)	75.1 (7.1)	0.044
Modified Frailty Index			
Frail	20 (18.2)	47 (26.0)	0.126
Cancer type (colon vs. rectal)			
Colon	76 (69.1)	118 (65.2)	0.494
Surgery type			
Open	41 (37.3)	78 (43.3)	0.505
Laparoscopic	65 (59.1)	98 (54.4)	
Robotic	4 (3.6)	4 (2.2)	
Intra-operative complication			
Yes	2 (1.8)	8 (4.4)	0.237
Stoma type			
No stoma	71 (64.6)	113 (62.4)	0.452
Loop ileostomy	27 (24.6)	39 (21.6)	
End colostomy	12 (10.9)	29 (16.0)	
Pathologic stage			
0	5 (4.6)	13 (7.2)	0.760
1	31 (28.2)	47 (26.0)	
2	39 (35.5)	55 (30.4)	
3	34 (30.9)	64 (35.4)	
4	1 (0.9)	2 (1.1)	
Negative margins			
Yes	107 (97.3)	174 (96.7)	0.773
Neoadjuvant			
Received	20 (18.2)	37 (20.4)	0.638
Adjuvant			
Received	42 (38.2)	53 (29.3)	0.148
Post-operative complication	28 (25.5)	56 (30.9)	0.317
Length of stay*	6.8 (4.9)	8.4 (8.2)	0.095
Discharge to a facility	9 (8.2)	43 (23.8)	0.003
In facility at follow-up	4 (3.6)	31 (17.1)	0.001
Readmission in 1 year	36 (32.7)	63 (34.8)	0.717

Demographics of surveyed patients and non-surveyed patients are also compared in Table 1. The surveyed group did not differ significantly from the non-surveyed group in regards to most demographics, cancer staging, surgery, or treatment-related variables. However, the surveyed group was slightly younger (mean: 73.2 and range: 65-89, compared to mean: 75.1 and range: 65-89 years; p = 0.044), was less likely to be discharged to a facility (8.2% compared to 23.8%; p = 0.003), and was more likely to be home at first follow-up (96.4% compared to 82.9%; p = 0.001). There was no significant difference in the number of frail patients by mFI in the survey group (18.2% compared to 26.0%; p = 0.126). Surveyed patients' Barthel Index at each time interval is displayed in Table 2.

**Table 2:** Patients at each Barthel Index level at each time point.

	Independent, n (%)	Slightly dependent, n (%)	Moderately dependent, n (%)	Severely dependent, n (%)
Pre-operative	61 (59.8)	16 (15.7)	25 (24.5)	0 (0)
6 months post-operative	46 (45.1)	16 (15.7)	37 (36.2)	3 (2.9)
1 year post-operative	47 (46.1)	18 (17.7)	32 (31.4)	5 (4.9)

Declining function at six months

Patients were categorized as having declining function if their Barthel Index grouping decreased by at least one category at six months post-operatively (Table 3). On univariate analysis, medical frailty based on mFI was not associated with the declining function (p = 0.281). Factors associated with declining function included race and pre-operative dependency based on Barthel Index (p = 0.035 and p = 0.006, respectively). However, on multivariable analysis accounting for age, race, gender, and readmission within one year, only pre-operative Barthel Index of moderate dependence was significantly associated with declining function at six months with an OR of 8.811 (CI: 1.824-42.552). Patients who were independent or slightly dependent pre-operatively did not experience significantly declining function six months after surgery.

**Table 3:** Factors associated with increasing frailty on Barthel Index at six months.

	Univariate analysis			Multivariate analysis	
	Barthel Index did not decline	Barthel Index declined	P-value	OR	95% CI
Sex					
Male	39 (50.7)	16 (48.5)	0.835		
Female	38 (49.4)	17 (51.5)		0.776	0.307-1.961
Age	73.2 (5.7)	73.3 (5.6)	0.881	0.996	0.917-1.081
Race					
White	64 (83.1)	29 (87.9)	0.035		
Black	13 (16.9)	2 (6.1)		4.373	0.874-21.874
Other	0 (0.0)	2 (6.1)			
Pathologic stage					
0	3 (3.9)	2 (6.1)	0.440		
1	20 (26.0)	11 (33.3)			
2	28 (36.4)	11 (33.3)			
3	26 (33.8)	8 (24.2)			
4	0 (0.0)	1 (3.0)			
Stoma					
No stoma	51 (66.2)	20 (60.6)	0.271		
Loop ileostomy	16 (20.8)	11 (33.3)			
End colostomy	10 (13.0)	2 (6.1)			
Procedure type					
Open	30 (39.0)	11 (33.3)	0.815		
Laparoscopic	44 (57.1)	21 (63.6)			
Robotic	3 (3.9)	1 (3.0)			
Cancer type					
Colon	56 (72.7)	20 (60.6)	0.207		
Rectal	21 (27.3)	13 (39.4)			
Intra-operative complication					
Yes	2 (2.6)	0 (0.0)	0.350		
Received neoadjuvant					
Yes	15 (19.5)	5 (15.2)	0.590		
No	62 (80.5)	28 (84.9)			
Received adjuvant					
Yes	32 (41.6)	10 (30.3)	0.307		
No	43 (55.8)	23 (69.7)			
Unknown	2 (2.6)	0 (0.0)			
Modified Frailty Index					
Frail	16 (20.8)	4 (12.1)	0.281		
Not frail	61 (79.2)	29 (87.9)			
Barthel Index pre-operatively					
Independent	39 (50.7)	25 (75.8)	0.006		
Slightly dependent	11 (14.3)	6 (18.2)		1.350	0.420-4.339
Moderately dependent	27 (35.1)	2 (6.1)		8.811	1.824-42.552
Post-operative complication					
Yes	22 (28.6)	6 (18.2)	0.252		
No	55 (71.4)	27 (81.8)			
Discharged to					
Home	43 (58.1)	16 (50.0)	0.742		
Home health/physical therapy	25 (33.8)	13 (40.6)			
Skilled nursing facility	6 (8.1)	3 (9.4)			
Readmission within 1 year					
Yes	29 (37.7)	7 (21.2)	0.092	1.411	0.493-4.040
No	48 (62.3)	26 (78.8)			
Length of stay	6.7 (4.5)	6.8 (5.3)	0.933		

Declining function at one year

Patients were categorized as having a declining function at one year if their Barthel Index level dropped by at least one category from their pre-operative score (Table 4). On univariate analysis, medical frailty based on mFI was not significantly associated with the declining function (p = 0.158). The presence of a stoma after surgery, rectal cancer, and receipt of neoadjuvant therapy were significantly associated with the declining function (p = 0.004, p = 0.008, and p = 0.012, respectively) on univariate analysis. On multivariable analysis accounting for age, gender, presence of a stoma, cancer type, receipt of neoadjuvant and mFI, only pre-operative Barthel Index of moderately dependent and black race were significantly associated with declining function with an OR of 17.504 (CI: 2.792-109.762) and 18.814 (CI: 1.547-228.767), respectively. Patients who were independent or slightly dependent pre-operatively did not experience significantly declining function.

**Table 4:** Factors associated with increasing frailty on Barthel Index at one year. * Numbers are given as mean and standard deviation in parentheses.

	Univariate analysis			Multivariate analysis	
	Barthel Index did not worsen (n = 80), n (%)	Barthel Index worsened (n = 30), n (%)	P-value	OR	95% CI
Sex					
Male	44 (55.0)	11 (36.7)	0.087		
Female	36 (45.0)	19 (63.3)		0.197	0.055-1.065
Age*	73.0 (5.6)	73.8 (5.9)	0.476	0.950	0.847-1.066
Race					
White	65 (81.3)	28 (93.3)	0.128		
Black	14 (17.5)	1 (3.3)		18.814	1.547-228.767
Other	1 (1.3)	1 (3.3)			
Pathologic stage					
0	5 (6.3)	0 (0.0)	0.230		
1	20 (25.0)	11 (36.7)			
2	26 32.5)	13 (43.3)			
3	28 (35.0)	6 (20.0)			
4	1 (1.3)	0 (0.0)			
Stoma					
No stoma	58 (72.5)	13 (43.3)	0.004		
Loop ileostomy	13 (16.3)	14 (46.7)		0.152	0.012-1.919
End colostomy	9 (11.3)	3 (10.0)		2.117	0.136-32.941
Procedure type					
Open	29 (36.3)	12 (40.0)	0.935		
Laparoscopic	48 (60.0)	17 (56.7)			
Robotic	3 (4.8)	1 (3.3)			
Cancer type					
Colon	61 (76.3)	15 (50.0)	0.008		
Rectal	19 (23.8)	15 (50.0)		0.810	0.145-4.522
Intra-operative complication					
Yes	1 (1.3)	1 (3.3)	0.466		
Received neoadjuvant					
Yes	10 (12.5)	10 (33.3)	0.012	0.192	0.035-1.959
No	70 (87.5)	20 (66.7)			
Received adjuvant					
Yes	32 (40.0)	10 (33.3)	0.653		
No	47 (58.8)	19 (63.3)			
Unknown	1 (1.3)	1 (3.3)			
Modified Frailty Index					
Frail	12 (15.0)	8 (26.7)	0.158	0.342	0.090-1.295
Not frail	68 (85.0)	22 (73.3)			
Barthel Index pre-operatively					
Independent	42 (52.5)	22 (73.3)	0.053		
Slightly dependent	12 (15.0)	5 (16.7)		2.270	0.499-10.334
Moderately dependent	26 (32.5)	3 (10.0)		17.504	2.792-109.762
Post-operative complication					
Yes	20 (25.0)	8 (26.7)	0.858		
No	60 (75.0)	22 (73.3)			
Discharged to					
Home	47 (61.8)	12 (40.0)	0.055		
Home health/physical therapy	25 (32.9)	13 (43.3)		1.325	0.193-9.097
Skilled nursing facility	4 (5.3)	5 (16.7)		0.154	0.012-1.895
Readmission within 1 year					
Yes	25 (31.3)	11 (36.7)	0.590		
No	55 (68.8)	19 (63.3)			
Length of stay*	6.6 (4.7)	7.2 (4.9)	0.225		

Failure to return to the baseline level of function

Eight patients were excluded from the subjective failure to return to baseline analysis because they were unsure of whether they had returned to baseline. Of the remaining 102 patients, 76 felt they did eventually return to their pre-operative baseline level of functioning, whereas 26 felt they never did. Univariate and multivariable analyses were performed to determine associations with subjective failure to return to baseline (Table 5). Neither mFI nor demographics were predictive of failure to return to baseline on univariate analysis. Pre-operative Barthel Index and discharge location were significantly associated with failure to return to baseline on univariate analysis (p = 0.031 and p = 0.015, respectively). Given the size of the study population, and the number of factors to be included in multivariate analysis, a backward stepwise regression was performed to further elucidate factors associated with return to baseline. Pre-operative Barthel Index was the only variable significantly associated with subjective failure to return to baseline (OR: 4.798, CI: 1.148-20.058) for slightly dependent and (OR: 4.188, CI: 1.105-15.875) moderately dependent patients.

**Table 5:** Factors associated with subjective return to baseline. * Numbers are given as mean and standard deviation in parentheses.

	Univariate analysis			Multivariate analysis	
	Returned to baseline (n = 76), n (%)	Did not return to baseline (n = 26), n (%)	P-value	OR	95% CI
Sex					
Male	36 (47.4)	12 (46.2)	0.915		
Female	40 (52.6)	14 (53.8)			
Age*	73.8 (5.6)	72.2 (6.1)	0.162	0.948	0.858-1.048
Race					
White	63 (82.9)	23 (88.5)	0.443		
Black	12 (15.8)	2 (7.7)			
Other	1 (1.3)	1 (3.8)			
Pathologic stage					
0	4 (5.3)	0 (0.0)	0.656		
1	21 (27.6)	9 (34.6)			
2	28 (36.8)	8 (30.8)			
3	22 (28.9)	9 (34.6)			
4	1 (1.3)	0 (0.0)			
Stoma					
No stoma	54 (71.1)	14 (53.8)	0.271		
Loop ileostomy	17 (22.4)	9 (34.6)			
End colostomy	5 (6.6)	3 (11.5)			
Procedure type					
Open	30 (39.5)	8 (30.8)	0.062	0.247	0.065-0.934
Laparoscopic	45 (59.2)	15 (57.7)			
Robotic	1 (1.3)	3 (11.5)		3.045	0.209-44.451
Cancer type					
Colon	57 (55.9)	15 (14.7)	0.095		
Rectal	19 (18.6)	11 (10.8)			
Intra-operative complication					
Yes	1 (1.32)	1 (3.85)	0.422		
Received neoadjuvant					
Yes	11 (14.5)	8 (30.8)	0.065	2.093	0.520-8.420
No	65 (85.5)	18 (69.2)			
Received adjuvant					
Yes	29 (38.2)	10 (38.5)	0.704		
No	45 (59.2)	16 (61.5)			
Unknown	2 (2.6)	0 (0.0)			
Modified Frailty Index					
Frail	12 (15.8)	7 (26.9)	0.208	2.473	0.625-9.790
Not frail	64 (84.2)	19 (73.1)			
Barthel Index pre-operatively					
Independent	51 (67.1)	10 (38.5)	0.031		
Slightly dependent	9 (11.8)	7 (26.9)		4.798	1.148-20.058
Moderately dependent	16 (21.1)	9 (34.6)		4.188	1.105-15.875
Post-operative complication					
Yes	17 (22.4)	9 (34.6)	0.216		
No	59 (77.6)	17 (65.4)			
Discharged to					
Home	48 (66.7)	9 (34.6)	0.015		
Home health/physical therapy	18 (25.0)	14 (53.8)		3.626	0.887-14.821
Skilled nursing facility	6 (8.3)	3 (11.5)		1.437	0.193-10.730
Readmission within 1 year					
Yes	21 (27.6)	10 (38.5)	0.300		
No	55 (72.4)	16 (61.5)			
Length of stay*	5.9 (3.6)	8.7 (6.6)	0.092	1.092	0.962-1.240

Time to return to baseline

**Figure 1:**
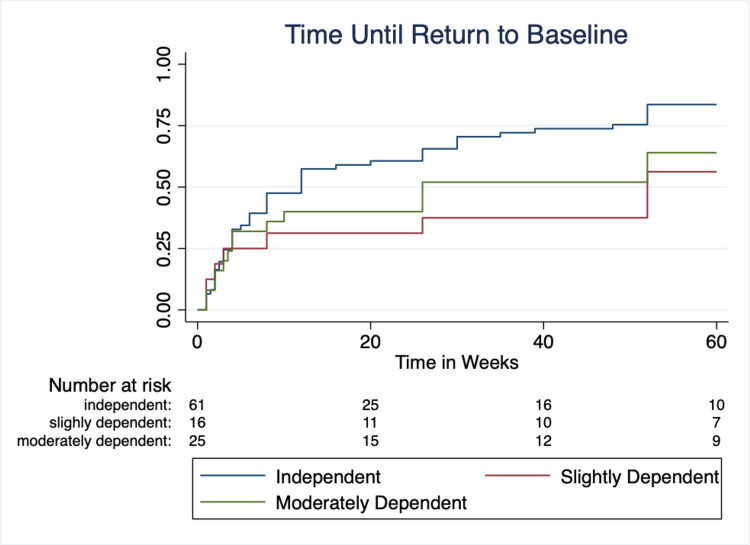
Kaplan-Meier curve demonstrating time to return to baseline.

The time to return to baseline depending on Barthel Index was estimated using a Kaplan-Meier curve and is shown in Figure 1. Differences between groups on Kaplan-Meier curves were evaluated with a log-rank test for statistical significance. Fewer patients that were dependent pre-operatively returned to baseline and took longer to do so on average (mean time to return to baseline was 15.1 weeks for independent, 21.9 weeks for slightly dependent, and 17.0 weeks for moderately dependent; p = 0.039).

## Discussion

This study indicates that medical frailty, as determined by the mFI, is not predictive of long-term functional outcomes in a population of geriatric colorectal cancer patients. In contrast, pre-operative functional frailty, as determined by Barthel Index, appeared to be predictive of functional outcomes at six months and one year. In addition, the Barthel Index was predictive of patients’ self-reported return to baseline function.

Most surgeons use medical data to evaluate a patient’s appropriateness for surgery, such as the American College of Surgeons National Surgical Quality Improvement Program (ACS NSQIP) calculator. However, given the predictive value of frailty assessments, ACS-NSQIP also recommends pre-operative screening for frailty in all geriatric patients undergoing colorectal surgery [25]. It is important that the screening tools provide information on the most meaningful outcomes to patients and surgeons. The modified frailty index has been shown to be associated with short-term morbidity and mortality, and like most risk calculators, is based primarily on medical comorbidities [4-8,26-30]. However, data from this study show that mFI is a poor predictor of long-term functional outcomes. This suggests that while indicators of medical frailty, such as mFI, are good predictors of short-term morbidity and mortality, they may not be predictive of long-term functional outcomes.

Pre-operative functional or physical frailty might be a better predictor of long-term functional impairment. Several previous studies have found evidence of functional decline following surgery, but have failed to show that frailty indicators, such as the ability to perform pre-operative ADLs are significantly associated with this decline [18-20]. In contrast, Finlayson et al. found that the functional deficit prior to colon surgery is associated with further functional decline one year after surgery [17]. Stabenau et al. demonstrated similar findings, actually mapping patients’ post-operative recovery course based on their level of functional deficit (“no disability” to “severe disability”). They found that pre-operative functional deficit directly correlated with the speed and degree of functional recovery after surgery [15]. Our results similarly indicate that pre-operative severity of dependency was associated with long-term functional decline, which strengthens the conclusion that pre-operative functional frailty is more predictive of long-term functional outcomes following surgery.

The long-term functional outcome may be a more important target for geriatric patients. Colorectal cancer is often curable with surgery, and prior studies have shown that without post-operative complications, cancer-related life expectancy is similar between older and younger patients [25]. However, in patients with a limited life expectancy, the functional return to baseline is a major factor in deciding to undergo surgery. Fried et al. demonstrated a majority of patients (88.8%) would accept even a high burden of treatment if they returned to their baseline. The same study also demonstrated that 74.4% of patients would not accept any treatment that resulted in significant functional impairment [2]. As a result, there is increased interest in predicting the functional recovery of geriatric patients, and developing effective interventions to improve their recovery [25]. Data from the current study show that 74.5% of all patients and 83.6% of functionally independent patients returned to baseline, which should provide some level of comfort and guidance to geriatric patients deciding to pursue extirpative surgery.

There are several important limitations to this study. First, there is no conclusive data on which measure is best used to determine frailty pre-operatively. As measures of frailty were not used prospectively, the authors selected measures that were appropriate for the retrospective collection. The retrospective nature of this study is also a limitation in terms of recall bias and patient dropout. While recall bias remains the most significant weakness of this study, the Barthel Index was selected to mitigate this risk given its ease in patient self-report. The Barthel Index is limited in that it does not have a separate scoring system for patients with an ostomy. Patients with a permanent ostomy were treated as if they were functionally independent in the area of bowel continence, so as not to bias their functional score. In addition, surgical resection of rectal cancer may result in urgency, which may negatively affect the incontinence score and overall Barthel Index. As a telephone survey, this study may also have limited access to certain frail patients such as nursing home residents or patients with dementia or other memory problems. Given the potential for recall bias and the risk for the potential cognitive decline since the time of surgery, patients were given the option to opt out of the survey at any point if unable to answer survey questions. In addition, patients were excluded if their family members felt they would not be able to participate due to cognitive decline. However, no formal evaluation of cognitive status was included as part of the study. While survey participants were overall similar to non-survey participants, significantly more non-participants were discharged to a facility and in a facility at follow-up, which may have increased bias. The power of this study was also limited and resulted in widened confidence intervals. Given the limitations of recall bias, access to patients at a nursing facility or with cognitive decline, and limited power, results must be interpreted with caution.

The results of this study help provide a framework for discussing pre-operative risk with patients prior to surgery with regards to frailty and help clarify the risk that medical and functional frailty impose. Medical frailty has been associated with poorer short-term post-operative outcomes, but this study appears to indicate that functional frailty may be a better predictor of long-term functional outcomes. Previous research indicates that the prospect of long-term functional decline often changes patients’ willingness to undergo treatment [2]. While previous studies have demonstrated improved outcomes when geriatricians are included in pre-operative planning, further research is needed to assess whether palliative care would be useful in cases where surgical management of colorectal cancer poses unacceptable risks of functional impairment [25]. Pre-habilitation programs have also shown promise to improve outcomes of frail or at-risk patients; however, further research is needed to elucidate the ideal pre-operative assessment of frailty and the ideal pre-habilitation program [25].

## Conclusions

Functional frailty, as measured by the Barthel Index, was better than medical frailty at predicting a patient’s long-term outcome in this study. Functional frailty prior to surgery was a risk for patients failing to return to baseline and declining function following surgery. While medical frailty, measured by mFI, may be useful for predicting the risk of peri-operative complications, it failed to predict long-term functional outcomes. These results appear to be consistent with prior research, but conclusions should be interpreted with caution due to the limitations of the study.

## Appendices

Addendum A

Survey questions

Before surgery

1. Were you able to cook and eat meals by yourself before surgery? 

Yes, some, not at all

2. Able to bathe independently before surgery?

Yes, no

3. Able to groom yourself independently before surgery?

Yes, no

4. Were you able to dress yourself before surgery?

Yes, some, not at all

5. Did you have trouble controlling your bowels before surgery? (Have accidents?)

Yes, some, not at all

6. Did you have trouble controlling your bladder before surgery? (Have accidents?)

Yes, some, not at all

7. Are you able to use the bathroom by yourself before surgery?

Yes, some, not at all

8. When getting out of bed, do you need some help before surgery?

Bedbound, a lot, a little, none

9. Were you able to walk 50 yards before surgery?

Yes, no, assistance, wheelchair

10. Were you able to climb a flight of stairs before surgery?

Yes, need help, no

Six months after surgery

1. Were you able to cook and eat meals by yourself six months after surgery?

Yes, some, not at all

2. Able to bathe independently six months after surgery?

Yes, no

3. Able to groom yourself independently six months after surgery?

Yes, no

4. Were you able to dress yourself six months after surgery?

Yes, some, not at all

5. Did you have trouble controlling your bowels six months after surgery? (Have accidents?)

Yes, some, not at all

6. Did you have trouble controlling your bladder six months after surgery? (Have accidents?)

Yes, some, not at all

7. Are you able to use the bathroom by yourself six months after surgery?

Yes, some, not at all

8. When getting out of bed, do you need some help six months after surgery?

Bedbound, a lot, a little, none

9. Were you able to walk 50 yards six months after surgery?

Yes, no, assistance, wheelchair

10. Were you able to climb a flight of stairs six months after surgery?

Yes, need help, no

One year after surgery (put it after every question)

1. Were you able to cook and eat meals by yourself one year after surgery?

Yes, some, not at all

2. Able to bathe independently one year after surgery?

Yes, no

3. Able to groom yourself independently one year after surgery?

Yes, no

4. Were you able to dress yourself one year after surgery?

Yes, some, not at all

5. Did you have trouble controlling your bowels one year after surgery? (Have accidents?)

Yes, some, not at all

6. Did you have trouble controlling your bladder one year after surgery? (Have accidents?)

Yes, some, not at all

7. Are you able to use the bathroom by yourself one year after surgery?

Yes, some, not at all

8. When getting out of bed, do you need some help one year after surgery?

Bedbound, a lot, a little, none

9. Were you able to walk 50 yards one year after surgery?

Yes, no, assistance, wheelchair

10. Were you able to climb a flight of stairs one year after surgery?

Yes, need help, no

11. Did you go to a rehab or assisted living facility after discharge? If yes, how long were you there?

12. Do you feel you were “back to your old self?” If so, when did you get there in relation to your surgery?
